# *Pneumocystis jirovecii* pneumonia in intensive care units: a multicenter study by ESGCIP and EFISG

**DOI:** 10.1186/s13054-023-04608-1

**Published:** 2023-08-24

**Authors:** Daniele Roberto Giacobbe, Silvia Dettori, Vincenzo Di Pilato, Erika Asperges, Lorenzo Ball, Enora Berti, Ola Blennow, Bianca Bruzzone, Laure Calvet, Federico Capra Marzani, Antonio Casabella, Sofia Choudaly, Anais Dartevel, Gennaro De Pascale, Gabriele Di Meco, Melissa Fallon, Louis-Marie Galerneau, Miguel Gallego, Mauro Giacomini, Adolfo González Sáez, Luise Hänsel, Giancarlo Icardi, Philipp Koehler, Katrien Lagrou, Tobias Lahmer, P. Lewis White, Laura Magnasco, Anna Marchese, Cristina Marelli, Mercedes Marín-Arriaza, Ignacio Martin-Loeches, Armand Mekontso-Dessap, Malgorzata Mikulska, Alessandra Mularoni, Anna Nordlander, Julien Poissy, Giovanna Russelli, Alessio Signori, Carlo Tascini, Louis-Maxime Vaconsin, Joel Vargas, Antonio Vena, Joost Wauters, Paolo Pelosi, Jean-Francois Timsit, Matteo Bassetti, JIR-ICU investigators (collaborators), Matteo Cerchiaro, Mario Zaccarelli, Chiara Robba, Denise Battaglini, Iole Brunetti, Filippo Del Puente, Sara Mora, Sofía de la Villa, Maricela Valerio, Patricia Muñoz, Gianmarco Lombardi, Melania Cesarano, Veronica Gennenzi, Philippe Meersseman, Greet Hermans, Alexander Wilmer, Keyvan Razazi, Guillaume Carteaux, Nicolas de Prost, Oliver A. Cornely, Danila Seidel, Ana Alastruey-Izquierdo, Jorge Garcia Borrega, Christine Bonnal, Etienne de Montmollin, Julien Dessajan, Mariaelena Ceresini, Francesco Mojoli, Ambra Vola, Cécile Garnaud, Emili Díaz, Oriol Gasch, Elena Prina, Sebastian Rasch, Miriam Dibos, Stefanie Haschka, the Critically Ill Patients Study Group of the European Society of Clinical Microbiology and Infectious Diseases (ESGCIP), and the Fungal Infection Study Group of the European Society of Clinical Microbiology and Infectious Diseases (EFISG)

**Affiliations:** 1 Department of Health Sciences (DISSAL), University of Genoa, Genoa, Italy; 2 Infectious Diseases Unit, San Martino Policlinico Hospital - IRCCS for Oncology and Neurosciences, L.go R. Benzi 10, 16132 Genoa, Italy; 3 Department of Surgical Sciences and Integrated Diagnostics (DISC), University of Genoa, Genoa, Italy; 4 Division of Infectious Diseases, Fondazione IRCCS Policlinico San Matteo, Pavia, Italy; 5 Anesthesia and Intensive Care, San Martino Policlinico Hospital - IRCCS for Oncology and Neurosciences, Genoa, Italy; 6 Assistance Publique - Hôpitaux de Paris, DMU Médecine, Service de Médecine Intensive Réanimation, Hôpital Henri Mondor, Hôpitaux Universitaires Henri Mondor, Créteil, France; 7 Department of Infectious Diseases, Karolinska University Hospital, Stockholm, Sweden; 8 Unit of Infectious Diseases, Department of Medicine Huddinge, Karolinska Institute, Stockholm, Sweden; 9 Hygiene Unit, San Martino Policlinico Hospital-IRCCS for Oncology and Neurosciences, Genoa, Italy; 10 Service de Médecine Intensive Réanimation, CHU de Clermont-Ferrand, Clermont-Ferrand, France; 11 Servizio di Anestesia e Rianimazione 1, Fondazione IRCCS Policlinico San Matteo, Pavia, Italy; 12 Microbiology Unit, Laboratory Department, Parc Taulí Hospital Universitari, Institut d’Investigació i Innovació Parc Taulí (I3PT-CERCA), Universitat Autònoma de Barcelona, Sabadell, Spain; 13 Inserm U1285, CHU Lille, CNRS, UMR 8576, UGSF, Unité de Glycobiologie Structurale Et Fonctionnelle, University of Lille, 59000 Lille, France; 14 Medical Intensive Care Unit, Grenoble Alpes University Hospital, Grenoble, France; 15 Dipartimento di Scienze Dell’emergenza, Anestesiologiche e Della Rianimazione, Fondazione Policlinico Universitario A. Gemelli IRCCS, Rome, Italy; 16 Università Cattolica del Sacro Cuore, Rome, Italy; 17 Public Health Wales Mycology Reference Laboratory, PHW Microbiology Cardiff, University Hospital of Wales, Heath Park, Cardiff, UK; 18 Respiratory Department, Parc Taulí Hospital Universitari, Institut d’Investigació i Innovació Parc Taulí (I3PT-CERCA), Universitat Autònoma de Barcelona, Sabadell, Spain; 19 Centro de Investigación Biomédica en Red de Enfermedades Respiratorias, Instituto de Salud Carlos III, Madrid, Spain; 20 Department of Informatics, Bioengineering, Robotics and System Engineering (DIBRIS), University of Genoa, Genoa, Italy; 21 Servicio de Microbiología Clínica y Enfermedades Infecciosas, Hospital General Universitario Gregorio Marañón, Madrid, Spain; 22 Instituto de Investigación Sanitaria Gregorio Marañón, CIBER Enfermedades Respiratorias-CIBERES, Facultad de Medicina, Universidad Complutense de Madrid, Madrid, Spain; 23 Department I of Internal Medicine, Excellence Centre for Medical Mycology (ECMM), Medical Faculty and University Hospital Cologne, University of Cologne, Cologne, Germany; 24 Medical Faculty and University Hospital Cologne, Cologne Excellence Cluster on Cellular Stress Responses in Aging-Associated Diseases (CECAD), University of Cologne, Cologne, Germany; 25 Department of Microbiology, Immunology and Transplantation, KU Leuven, Leuven, Belgium; 26 Department of Laboratory Medicine and National Reference Center for Mycosis, University Hospitals Leuven, Leuven, Belgium; 27 Department of Internal Medicine II, Klinikum Rechts der Isar, School of Medicine, Technical University of Munich, Munich, Germany; 28 Division of Infection and Immunity, Cardiff University Centre for Trials Research, Heath Park, Cardiff, UK; 29 UO Microbiologia, San Martino Policlinico Hospital - IRCCS for Oncology and Neurosciences, Genoa, Italy; 30 Facultad de Medicina, Universidad Complutense de Madrid, Madrid, Spain; 31 Department of Intensive Care Medicine, Multidisciplinary Intensive Care Research Organization (MICRO), Dublin, Leinster Ireland; 32 Pulmonary Intensive Care Unit, Respiratory Institute, Hospital Clinic of Barcelona, IDIBAPS (Institut d’Investigacions Biomèdiques August Pi I Sunyer), University of Barcelona, CIBERES, Barcelona, Spain; 33 Groupe de Recherche Clinique CARMAS, Faculté de Santé de Créteil, IMRB, Creteil, Île-de-France France; 34 INSERM, Creteil, Île-de-France France; 35 Unit of Infectious Diseases, ISMETT-IRCCS Istituto Mediterraneo per i Trapianti e Terapie ad Alta Specializzazione, Palermo, Italy; 36 Department of Intensive Care Medicine, Critical Care Center, CHU Lille, 59000 Lille, France; 37 Section of Biostatistics, Department of Health Sciences (DISSAL), University of Genoa, Genoa, Italy; 38 Infectious Diseases Clinic, Azienda Sanitaria Universitaria del Friuli Centrale (ASUFC), Udine, Italy; 39 Department of Medical Area (DAME), University of Udine, Udine, Italy; 40 Medical and Infectious Diseases ICU, APHP, Bichat Hospital, Paris, France; 41 Medical Intensive Care Unit, University Hospitals Leuven, Leuven, Belgium; 42 INSERM, IAME, Université Paris Cité, Paris, France

**Keywords:** *Pneumocystis*, PCR, Pneumonia, ICU, Diagnosis, Biomarker, Serum β-D-Glucan

## Abstract

**Background:**

*Pneumocystis jirovecii* pneumonia (PJP) is an opportunistic, life-threatening disease commonly affecting immunocompromised patients. The distribution of predisposing diseases or conditions in critically ill patients admitted to intensive care unit (ICU) and subjected to diagnostic work-up for PJP has seldom been explored.

**Materials and methods:**

The primary objective of the study was to describe the characteristics of ICU patients subjected to diagnostic workup for PJP. The secondary objectives were: (i) to assess demographic and clinical variables associated with PJP; (ii) to assess the performance of *Pneumocystis* PCR on respiratory specimens and serum BDG for the diagnosis of PJP; (iii) to describe 30-day and 90-day mortality in the study population.

**Results:**

Overall, 600 patients were included in the study, of whom 115 had presumptive/proven PJP (19.2%). Only 8.8% of ICU patients subjected to diagnostic workup for PJP had HIV infection, whereas hematological malignancy, solid tumor, inflammatory diseases, and solid organ transplants were present in 23.2%, 16.2%, 15.5%, and 10.0% of tested patients, respectively. In multivariable analysis, AIDS (odds ratio [OR] 3.31; 95% confidence interval [CI] 1.13–9.64, *p* = 0.029), non-Hodgkin lymphoma (OR 3.71; 95% CI 1.23–11.18, *p* = 0.020), vasculitis (OR 5.95; 95% CI 1.07–33.22, *p* = 0.042), metastatic solid tumor (OR 4.31; 95% CI 1.76–10.53, *p* = 0.001), and bilateral ground glass on CT scan (OR 2.19; 95% CI 1.01–4.78, *p* = 0.048) were associated with PJP, whereas an inverse association was observed for increasing lymphocyte cell count (OR 0.64; 95% CI 0.42–1.00, *p* = 0.049). For the diagnosis of PJP, higher positive predictive value (PPV) was observed when both respiratory *Pneumocystis* PCR and serum BDG were positive compared to individual assay positivity (72% for the combination vs. 63% for PCR and 39% for BDG). Cumulative 30-day mortality and 90-day mortality in patients with presumptive/proven PJP were 52% and 67%, respectively.

**Conclusion:**

PJP in critically ill patients admitted to ICU is nowadays most encountered in non-HIV patients. Serum BDG when used in combination with respiratory *Pneumocystis* PCR could help improve the certainty of PJP diagnosis.

**Supplementary Information:**

The online version contains supplementary material available at 10.1186/s13054-023-04608-1.

## Introduction

*Pneumocystis jirovecii* pneumonia (PJP) was most commonly described as an opportunistic, life-threatening disease in patients with human immunodeficiency virus (HIV) infection [1, 2]. However, due to the increased use of immunosuppressants, biologic agents, and corticosteroids for treating diseases such as inflammatory diseases, hematological malignancies, and solid neoplasms, novel populations at risk of PJP have emerged over the last decades, with reported mortality ranging from 33 to 69% [3–7].

Patients with severe PJP often require intensive care management. Polymerase chain reaction (PCR) targeting *Pneumocystis* DNA from respiratory samples and serum (1,3)-β-D-glucan (BDG) are widely used to support the diagnosis of PJP in these patients, despite the heterogeneity in assay design and positivity threshold [8–12]. The distribution of predisposing diseases/conditions in critically ill patients admitted to intensive care unit (ICU) and subjected to diagnostic workup for PJP, or diagnosed with PJP, has seldom been explored, and usually in small, single center cohorts limiting confidence in findings [13].

The present multinational, multicenter, retrospective study was conducted to describe the demographic and clinical characteristics of critically ill patients admitted to ICU and undergoing a PJP diagnostic workup, in order to understand the current distribution of predisposing diseases and conditions both in patients evaluated for PJP and in those with a PJP diagnosis. The performance of respiratory *Pneumocystis* PCR and serum BDG for the diagnosis of PJP in the study population was also explored as a secondary aim.

## Materials and methods

### Study setting and objectives

The present retrospective, multicenter, multinational study, coordinated by San Martino Polyclinic Hospital in Genoa, Italy, was conducted in 8 different countries for a total of 18 participating centers (five in France, five in Italy, two in Germany, two in Spain, one in Belgium, one in Ireland, one in Sweden, and one in UK). The retrospective study period was from 1 January 2016 to 31 December 2020. Consecutive ICU patients with radiographically documented pneumonia who underwent *Pneumocystis* polymerase chain reaction (PCR) testing on respiratory specimens (sputum, tracheal aspirate, and/or bronchoalveolar lavage fluid) and/or serum (1,3)-β-D-glucan (BDG) within a diagnostic workup for *Pneumocystis jirovecii* pneumonia (PJP) were included in the study. Centers could opt for participating only for most recent years of the study period, provided all consecutive patients meeting inclusion criteria were included for the selected years. Exclusion criteria were: (1) age < 18 years; (2) patient already included in the study. The primary objective of the study was to describe the characteristics of ICU patients suspected to have PJP. The secondary objectives were: (1) to assess demographic and clinical variables associated with diagnosis of PJP; (2) to assess the performance of respiratory *Pneumocystis* PCR and serum BDG for the diagnosis of PJP; (3) to describe 30-day and 90-day mortality in the study population. The study was approved by the ethics committee of the coordinating center (Liguria Region Ethics Committee, N. Registro CER Liguria 305/2021). Informed consent was waived due to the retrospective nature of the study. The other participating centers followed the local ethical requirements.

### Definitions

For patients with available microscopy results, proven PJP was defined as detection of *Pneumocystis* in respiratory specimens via conventional or immunofluorescence staining, according to EORTC/MSGERC definitions [14]. In line with the specific aims of the present study, we did not employ the EORTC/MSGERC criteria for defining probable PJP [14]. Instead, the diagnostic categories for PJP in the absence of a proven diagnosis were defined as “presumptive PJP”, “no PJP”, or “PJP diagnosis inconclusive”, based on independent review of completed electronic case report forms (eCRF) for each patient by two independent medical investigators (D.R.G. and S.D.), with cases of disagreement being resolved by a third medical investigator (A.V.). Although this approach is not standardized and is not exempt from biases (see study limitations in the discussion), the use of the “probable” diagnostic category as per EORTC/MSGERC criteria was deemed as unsuitable for the specific aims of the present study, for the following reasons: (i) it was frequently not possible to define probable PJP retrospectively due to the lack of all the required data for categorization (e.g., presence, dosage, and length of steroid treatment); and, most importantly, (ii) *Pneumocystis* PCR and/or serum BDG are necessary mycological criteria for defining probable PJP according to EORTC/MSGERC criteria, thereby resulting in a significant incorporation bias when assessing the diagnostic performance of either of the two markers [14]. Certainly, our approach does not eliminate incorporation bias since the results of the two markers also influenced the independent categorization as presumptive PJP by the investigators. However, categorization was conceived on a more global assessment of the clinical picture and disease course (i.e., concomitant presence of alternative causative agents of interstitial pneumonia, response to treatment for PJP and for concomitant infections). A detailed list of the diagnostic tests employed in the different participating centers is available as Additional file (Additional file 1: Table S1).

### Data collection

Data were uploaded by the local investigator on an electronic case report form (eCRF) specifically designed for the present study and reviewed by S.D. and F.D.P., with real-time generation of pertinent queries that were resolved by local investigators [15]. The following data related to demographics and medical history were collected from the patients’ medical charts: age in years; sex, Charlson Comorbidity Index [16]; HIV infection; presence of acquired immune deficiency syndrome (AIDS); presence of hematological malignancies; previous hematopoietic stem cell transplantation (HSCT); previous solid organ transplantation (SOT); presence of inflammatory diseases; presence of solid tumor; chemotherapy and/or radiotherapy in the previous 30 days, presence of chronic pulmonary diseases, presence of chronic kidney disease (defined as glomerular filtration rate < 60 mL/min), presence of chronic liver disease (defined histologically as liver cirrhosis or in presence of a clinical diagnosis supported by laboratory, endoscopy, and radiologic findings [17]); New York Heart Association (NYHA) score; previous intravenous immunoglobulin therapy (within 30 days), previous blood transfusions (within 30 days); previous albumin therapy (within 30 days); previous major surgery (within 30 days); receipt of PJP prophylaxis. Besides information on serum BDG and respiratory *Pneumocystis* PCR results, the following data were also collected at time of PJP suspicion: characteristics of lung lesion/s at computerized tomography; presence and type of concomitant infections; blood leukocyte count; blood lymphocyte count, blood CD4 + T lymphocyte count; blood neutrophil count; serum C-reactive protein (CRP); serum procalcitonin (PCT); presence of invasive mechanical ventilation; presence of acute respiratory distress syndrome (ARDS) [18]; presence of septic shock [19]; sequential organ failure assessment (SOFA) score [20]; receipt of continuous renal replacement therapy (CRRT); receipt of PJP therapy.

### Statistical analysis

The primary study objective was to describe the characteristics of ICU patients suspected to have PJP. To this aim, proportions for categorical variables and median values for continuous variables are presented descriptively, both in the entire cohort and stratified according to PJP diagnosis. To assess factors associated with PJP, demographic and clinical variables were first tested for their possible association with PJP in univariable logistic regression models. All variables potentially associated with presumptive/proven PJP by univariable analyses (*p* < 0.10) were included in an initial multivariable logistic regression model and further selected for inclusion in a final multivariable model (model A) using a stepwise, backward procedure. In addition, variables included in model A were also included in a second generalized linear mixed model with logit as the link function (model B), which also included center as a random effect. The diagnostic performance of respiratory *Pneumocystis* PCR and serum BDG for the diagnosis of PJP (setting either presumptive/proven PJP or only proven PJP as the diagnostic references) was assessed in terms of sensitivity, specificity, positive predictive value (PPV), negative predictive value (NPV), positive likelihood ratio (LR+), and negative likelihood ratio (LR−). A serum BDG value equal or above the manufacturer cut-off (according to the type of employed assay) was defined as the criterion for serum BDG positivity. For the molecular detection of *Pneumocystis*, any positive result (irrespective of the Ct value) was considered significant for respiratory *Pneumocystis* PCR positivity. For available paired data, the possible correlation between serum BDG values and Ct values from *Pneumocystis* PCR assays performed on different specimens (sputum, tracheal aspirate, or BALF) was assessed by measuring Spearman’s correlation coefficient. Survival up to either 30-day or 90-day was descriptively summarized through Kaplan–Meier curves and compared between patients with PJP and without PJP using the log-rank test. Statistical analyses were performed with R Statistical Software (version 3.6.0, R Foundation for Statistical Computing, Vienna, Austria) and SPSS Statistics (version 29.0, IBM Corp., Armonk, NY, US).

## Results

**Fig. 1:**
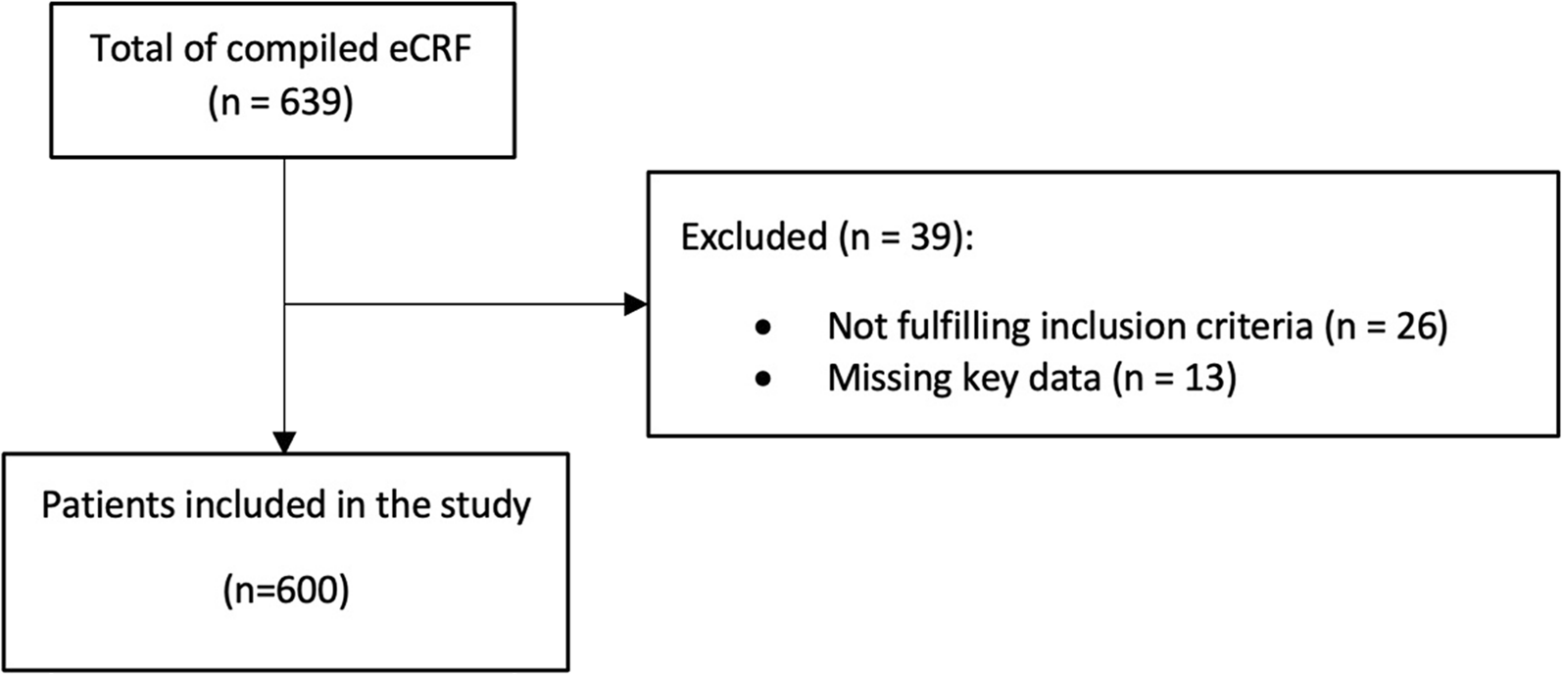
Flow diagram of the patients’ inclusion process. eCRF, electronic case report forms. Missing key data was defined as unavailability of information regarding both *Pneumocystis* polymerase chain reaction results and serum β-D-glucan results

Overall, 600 patients were included in the study (Fig. 1). Of them, 115 were classified as presumptive/proven PJP (19.2%), and 444/600 were classified as no PJP (74%). The remaining 41/600 patients (6.8%) were classified as “PJP diagnosis inconclusive”. Among patients with PJP, 31/115 (27.0%) and 84/115 (73.0%) were classified as proven PJP and presumptive PJP, respectively. The baseline demographic and clinic characteristics were similar between patients with proven PJP and presumptive PJP (Additional file 1: Table S2). As shown in Table 1, only 8.8% of patients subjected to diagnostic workup for PJP had HIV infection, whereas hematological malignancy, solid tumors, inflammatory diseases, and solid organ transplants were present in 23.2%, 16.2%, 15.5%, and 10.0% of patients, respectively. Concomitant coronavirus disease 2019 was present in 75 patients (12.6%). Only 3.8% of patients were under PJP prophylaxis (in all cases with trimethoprim/sulfamethoxazole) at the time of BDG/PCR testing. The median SOFA score at the time of the BDG/PCR testing was 6 (interquartile range 4–9), and as many as 28.9% of patients had septic shock. The frequency of presumptive/proven PJP by baseline condition/disease was 42.3% (22/52) in patients with HIV infection, and 55.9% (19/34) among HIV infected patients with AIDS, while among patients with hematological malignancy, solid tumor, inflammatory diseases, and solid organ transplants it was 25.9% (36/139), 28.9% (28/97), 18.3% (17/93), and 10.0% (6/60), respectively. Of note, the frequency of presumptive/proven PJP in our study population was as high as 47.1% (16/34) and 40.0% (14/35) in patients with non-Hodgkin lymphoma and metastatic solid neoplasms, respectively.

**Table 1:** Baseline characteristics of the study population

Variable*	Total n = 600 (100)	Patients with presumptive/proven PJP n = 115 (19.2)	Patients without PJP n = 444 (74.0)	Patients with PJP diagnosis inconclusive n = 41 (6.8)
*Demographic*				
Age in years, median (IQR)	61 (52–70)	60 (49–70)	62 (53–71)	61 (56–69)
Female sex	203 (33.8)	35 (30.4)	156 (35.1)	12 (29.3)
*Medical history*				
HIV infection (missing = 10)	52 (8.8)	22 (19.5)	26 (6.0)	4 (9.8)
AIDS (missing = 12)	34 (5.8)	19 (17.0)	12 (2.8)	3 (7.3)
Solid organ transplant (missing = 3)	60 (10.0)	6 (5.2)	47 (10.7)	7 (17.5)
Liver	15 (2.5)	0 (0)	14 (3.2)	1 (2.5)
Kidney	27 (4.5)	5 (4.3)	19 (4.3)	3 (7.5)
Lung	9 (1.5)	1 (0.9)	7 (1.6)	1 (2.5)
Heart	9 (1.5)	0 (0)	7 (1.6)	2 (5.0)
Other	0 (0)	0 (0)	0 (0)	0 (0)
Hematological malignancy (missing = 2)	139 (23.2)	36 (31.3)	93 (21.0)	10 (25.0)
AML	43 (7.2)	4 (3.4)	38 (8.6)	1 (2.5)
ALL	9 (1.5)	3 (2.6)	6 (1.4)	0 (0)
Hodgkin lymphoma	8 (1.3)	2 (1.7)	5 (1.1)	1 (2.5)
Non-Hodgkin lymphoma	34 (5.7)	16 (13.9)	13 (2.9)	5 (12.5)
Other	42 (7.0)	8 (6.9)	31 (7.0)	3 (7.5)
HSCT (missing = 4)	57 (9.6)	12 (10.4)	42 (9.5)	3 (30.0)
Inflammatory disease (missing = 3)	93 (15.5)	17 (14.8)	63 (14.3)	13 (31.7)
Rheumatoid arthritis	15 (2.5)	3 (2.6)	9 (2.0)	3 (7.3)
Systemic lupus erythematosus	8 (1.3)	1 (0.9)	7 (1.6)	0 (0)
Polymyositis-dermatomyositis	7 (1.2)	0 (0)	5 (1.1)	2 (5.0)
Inflammatory bowel disease	4 (0.7)	2 (1.7)	2 (0.5)	0 (0)
Scleroderma	5 (0.8)	0 (0)	3 (0.7)	2 (5.0)
Vasculitis	9 (1.5)	4 (3.4)	5 (1.1)	0 (0)
Mixed connective tissue disease	8 (1.3)	1 (0.9)	6 (1.4)	1 (2.4)
Autoimmune hepatitis	2 (0.3)	1 (0.9)	1 (0.2)	0 (0)
Sarcoidosis	4 (0.7)	1 (0.9)	3 (0.7)	0 (0)
Autoimmune hemolytic anemia	3 (0.5)	1 (0.9)	2 (0.5)	0 (0)
Myasthenia gravis	5 (0.8)	0 (0)	5 (1.1)	0 (0)
Other	23 (3.9)	3 (2.6)	15 (3.4)	5 (12.2)
Solid tumor (missing = 3)	97 (16.2)	28 (24.6)	63 (14.2)	6 (15.0)
Metastatic solid tumor (missing = 6)	35 (5.9)	14 (12.6)	20 (4.5)	1 (2.5)
COPD (missing = 9)	68 (11.5)	11 (9.6)	51 (11.7)	6 (15.0)
*Chronic pulmonary diseases other than COPD*				
(missing = 12)	64 (10.8)	9 (8.0)	48 (11.0)	7 (17.9)
Asthma	16 (2.7)	2 (1.8)	12 (2.8)	2 (5.1)
Cystic fibrosis	9 (1.5)	1 (0.9)	8 (1.8)	0 (0)
Interstitial lung disease/pulmonary fibrosis	20 (3.4)	2 (1.8)	13 (3.0)	5 (12.8)
Other	19 (3.2)	4 (3.5)	15 (3.4)	0 (0)
Chronic kidney disease (missing = 7)	79 (13.3)	13 (11.4)	58 (13.2)	8 (20.0)
Chronic liver disease (missing = 6)	55 (9.3)	9 (7.9)	44 (10.0)	2 (5.0)
NYHA score > 2 (missing = 42)	84 (15.1)	13 (14.1)	65 (15.2)	6 (15.8)
Age-adjusted Charlson score, median (IQR)	4 (2–6)	4 (3–7)	4 (2–5)	4 (2–5)
Previous major surgery (within 30 days) (missing = 14)	56 (9.5)	6 (5.4)	48 (11.0)	2 (5.0)
Previous chemotherapy (within 30 days) (missing = 6)	121 (20.4)	39 (34.5)	72 (16.4)	10 (24.4)
Previous radiotherapy (within 30 days) (missing = 8)	19 (3.2)	10 (8.8)	8 (1.8)	1 (2.5)
Previous IVIG therapy (within 30 days) (missing = 24)	27 (4.7)	8 (7.5)	17 (3.9)	2 (5.3)
Previous albumin therapy (within 30 days)				
(missing = 43)	57 (10.2)	7 (6.6)	50 (11.9)	0 (0)
*Previous blood transfusions (within 30 days)*				
(missing = 46)	108 (19.5)	21 (20.0)	84 (20.1)	3 (9.7)
PJP prophylaxis (missing data = 25)	22 (3.8)	1 (1.1)	20 (4.5)	1 (2.4)
*Clinical and laboratory data at the time of PJP diagnostic workup*				
Length of hospital stay in days, median (IQR)				
(missing = 44)	8 (2–19)	3 (1–10)	9 (3–20)	6 (1–16)
*Length of ICU stay in days, median (IQR)*				
(missing = 19)	1 (0–6)	0 (-1 to 1)	2 (0–8)	0 (-3 to 1)
ARDS (missing = 37)	287 (50.9)	59 (57.8)	222 (52.1)	6 (17.1)
Invasive mechanical ventilation (missing = 11)	313 (53.1)	45 (40.2)	255 (58.5)	13 (31.7)
SOFA score, median (IQR) (missing = 4)	6 (4–9)	8 (4–10)	7 (4–9)	4 (3–5)
Septic shock (missing = 32)	164 (28.9)	29 (29.0)	130 (30.9)	5 (13.2)
CRRT (missing = 28)	62 (10.8)	8 (7.5)	53 (12.3)	1 (2.8)
CT scan performed (missing = 18)	329 (56.5)	69 (62.2)	230 (53.5)	30 (73.2)
No ground-glass opacities	23 (3.9)	0 (0)	23 (5.3)	0 (0)
Unilateral ground-glass opacities	8 (1.4)	3 (2.7)	4 (0.9)	1 (2.4)
Bilateral ground-glass opacities	86 (14.8)	22 (19.8)	46 (10.7)	18 (43.9)
Unilateral ground-glass and consolidations	20 (3.4)	1 (0.9)	19 (4.4)	0 (0)
Bilateral ground-glass and consolidations	185 (31.8)	39 (35.1)	136 (31.6)	10 (24.4)
Blood neutrophil count in cells × 10^–3^/mm^3^, median (IQR) (missing = 63)	7.2 (4.1–12.2)	6 (3.2–10.8)	7.7 (4.5–13.1)	6.0 (4.0–9.7)
Blood lymphocyte count in cells × 10^–3^/mm^3^, median (IQR) (missing = 120)	0.6 (0.3–1.1)	0.5 (0.2–0.9)	0.7 (0.4–1.2)	0.73 (0.5–1.2)
Serum CRP in mg/L, median (IQR) (missing = 49)	96 (23–187)	115.5 (42.3–217.5)	83 (20–179)	127 (36.5–193.0)
Serum PCT in ng/mL, median (IQR) (missing = 179)	0.86 (0.2–4.4)	0.4 (0.2–1.1)	1 (0.3–5.7)	0.4 (0.1–6.1)

AIDS: Acquired immune deficiency syndrome; ALL: Acute lymphocytic leukemia; AML: Acute myeloid leukemia; ARDS: Acute respiratory distress syndrome; COPD: Chronic obstructive pulmonary disease; CRRT: Continuous renal replacement therapy; CRP: C-reactive protein; CT: Computer tomography; HIV: Human immunodeficiency virus; HSCT: Hematopoietic stem cell transplantation; ICU: Intensive care unit; IQR: interquartile range; IVIG: Intravenous immunoglobulin; NYHA: New York Heart Association; PCT: Procalcitonin; PJP: *Pneumocystis jirovecii* pneumonia; SOFA: Sequential Organ Failure Assessment

*Data reported as no. (%) unless otherwise indicated. Number of missing values, impacting denominator and frequency calculation, are reported in parenthesis for each variable

The results of univariable and multivariable analyses of factors associated with presumptive/proven PJP, conducted in 559 patients (after the exclusion of 41/600 patients with inconclusive PJP diagnosis) are available in Tables 2 and 3, respectively. As shown in Table 2, HIV infection, AIDS, hematological malignancy, non-Hodgkin lymphoma, solid tumor, metastatic solid tumor, Charlson Comorbidity Index, previous chemotherapy, previous radiotherapy, increasing serum CRP values, and bilateral ground-glass opacities were associated with PJP in univariable analysis. Conversely, an inverse association with PJP was observed for increasing length of hospital and ICU stay before PJP testing, invasive mechanical ventilation, increasing lymphocyte cell count, and increasing neutrophil cell count. In multivariable model A (Table 3), AIDS (odds ratio [OR] 3.31; 95% CI 1.13–9.64, *p* = 0.029), non-Hodgkin lymphoma (OR 3.71; 95% CI 1.23–11.18, *p* = 0.020), vasculitis (OR 5.95; 95% CI 1.07–33.22, *p* = 0.042) metastatic solid tumor (OR 4.31; 95% CI 1.76–10.53, *p* = 0.001) and bilateral ground glass on CT scan (OR 2.19; 95% CI 1.01–4.78, *p* = 0.048) retained an association with PJP, whereas an inverse association with PJP was retained for invasive mechanical ventilation (OR 0.43; 95% CI 0.24–0.80, *p* = 0.007), and increasing lymphocyte cell count (OR 0.64; 95% CI 0.42–1.00, *p* = 0.049). The results of the additional multivariable model B including center as a random effect were in line with the directions of effects observed in model A (Table 3).

**Table 2:** Univariable analysis of factors associated with presumptive/proven PJP

Variable*	Total n = 559 (100)	Patients with presumptive/proven PJP n = 115 (20.6)	Patients without PJP n = 444 (79.4)	OR (95% CI)	*P*
*Demographic*					
Age in years, median (IQR)	61 (52–70)	60 (49–70)	62 (53–71)	0.99 (0.98–1.00)	0.15
Female sex	191 (34.2)	35 (30.4)	156 (35.1)	0.81 (0.52–1.26)	0.34
*Medical history*					
HIV infection (missing = 10)	48 (8.7)	22 (19.5)	26 (6.0)	3.81 (2.07–7.03)	< 0.001
AIDS (missing = 12)	31 (5.7)	19 (17.0)	12 (2.8)	7.20 (3.38–15.35)	< 0.001
Solid organ transplant (missing = 2)	53 (9.5)	6 (5.2)	47 (10.6)	0.46 (0.19–1.11)	0.08
Liver	14 (2.5)	0 (0)	14 (3.2)		§
Kidney	24 (4.3)	5 (4.3)	19 (4.3)	1.02 (0.37–2.78)	0.97
Lung	8 (1.4)	1 (0.9)	7 (1.6)	0.55 (0.07–4.5)	0.58
Heart	7 (1.3)	0 (0)	7 (1.6)		§
Other	0 (0)	0 (0)	0 (0)		
Hematological malignancy (missing = 1)	129 (23.1)	36 (31.3)	93 (21.0)	1.72 (1.09–2.71)	0.02
AML	42 (7.5)	4 (3.5)	38 (8.6)	0.39 (0.14–1.10)	0.075
ALL	9 (1.6)	3 (2.6)	6 (1.4)	1.96 (0.48–7.94)	0.35
Hodgkin lymphoma	7 (1.3)	2 (1.7)	5 (1.1)	1.55 (0.30–8.11)	0.60
Non**-**Hodgkin lymphoma	29 (5.2)	16 (10.3)	13 (2.9)	5.36 (2.50–11.50)	< 0.001
Other	39 (7.0)	8 (6.9)	31 (7.0)	0.99 (0.45–2.23)	0.99
HSCT (missing = 3)	54 (9.7)	12 (10.4)	42 (9.5)	1.24 (0.63–2.45)	0.54
Inflammatory disease (missing = 3)	80 (14.4)	17 (14.8)	63 (14.3)	1.04 (0.58–1.86)	0.89
Rheumatoid Arthritis	12 (2.2)	3 (2.6)	9 (2.0)	1.30 (0.35–4.86)	0.70
Systemic Lupus Erythematosus	8 (1.4)	1 (0.9)	7 (1.6)	0.55 (0.07–4.50)	0.58
Polymyositis-Dermatomyositis	5 (0.9)	0 (0)	5 (1.1)		§
Inflammatory bowel disease	4 (0.7)	2 (1.7)	2 (0.5)	3.91 (0.55–28.07)	0.18
Scleroderma	3 (0.5)	0 (0)	3 (0.7)		§
Vasculitis	9 (1.6)	4 (3.5)	5 (1.1)	3.16 (0.84–11.98)	0.09
Mixed Connective Tissue Disease	7 (1.3)	1 (0.9)	6 (1.4)	0.64 (0.08–5.37)	0.68
Autoimmune hepatitis	2 (0.4)	1 (0.9)	1 (0.2)	3.87 (0.24–62.60)	0.34
Sarcoidosis	4 (0.7)	1 (0.9)	3 (0.7)	1.29 (0.13–12.51)	0.83
Autoimmune hemolytic anemia	3 (0.5)	1 (0.9)	2 (0.5)	1.94 (0.17–21.57)	0.59
Myasthenia gravis	5 (0.9)	0 (0)	5 (1.1)		§
Other	18 (3.2)	3 (2.6)	15 (3.4)	0.77 (0.22–2.69)	0.68
Solid tumor (missing = 2)	91 (16.3)	28 (24.6)	63 (14.2)	1.96 (1.19–3.25)	0.009
Metastatic solid tumor	34 (6.1)	14 (12.6)	20 (4.5)	3.05 (1.49–6.26)	0.002
COPD (missing = 8)	62 (11.3)	11 (9.6)	51 (11.7)	0.81 (0.41–1.61)	0.54
Chronic pulmonary diseases other than COPD (missing = 10)	57 (10.4)	9 (8.0)	48 (11.0)	0.70 (0.33–1.47)	0.35
Asthma	14 (2.6)	2 (1.8)	12 (2.8)	0.64 (0.14–2.89)	0.56
Cystic fibrosis	9 (1.6)	1 (0.9)	8 (1.8)	0.48 (0.06–3.86)	0.49
Interstitial lung disease/pulmonary fibrosis	15 (2.7)	2 (1.8)	13 (3.0)	0.59 (0.13–2.64)	0.49
Other	19 (3.5)	4 (3.5)	15 (3.4)	1.03 (0.34–3.17)	0.96
Chronic kidney disease (missing = 6)	71 (12.8)	13 (11.4)	58 (13.2)	0.85 (0.45–1.60)	0.61
Chronic liver disease (missing = 5)	53 (9.6)	9 (7.9)	44 (10.0)	0.77 (0.37–1.63)	0.49
NYHA score > 2 (missing = 39)	78 (15.0)	13 (14.1)	65 (15.2)	0.92 (0.48–1.75)	0.79
Age-adjusted Charlson score, median (IQR)	4 (2–6)	4 (3–7)	4 (2–5)	1.14 (1.06–1.27)	< 0.001
Previous major surgery (within 30 days) (missing = 13)	54 (9.8)	6 (5.4)	48 (11.0)	0.46 (0.19–1.11)	0.08
Previous chemotherapy (within 30 days) (missing = 6)	111 (20.1)	39 (34.5)	72 (16.4)	2.69 (1.70–4.28)	< 0.001
Previous radiotherapy (within 30 days) (missing = 7)	18 (3.3)	10 (8.8)	8 (1.8)	5.23 (2.01–13.58)	< 0.001
Previous IVIG therapy (within 30 days) (missing = 21)	25 (4.6)	8 (7.5)	17 (3.9)	1.97 (0.83–4.69)	0.13
Previous albumin therapy (within 30 days) (missing = 34)	57 (10.9)	7 (6.6)	50 (11.9)	0.52 (0.23–1.19)	0.12
Previous blood transfusions (within 30 days) (missing = 36)	105 (20.1)	21 (20.0)	84 (20.1)	0.99 (0.58–1.70)	0.98
PJP prophylaxis (missing data = 25)	21 (3.9)	1 (1.1)	20 (4.5)	0.24 (0.03–1.80)	0.16
*Clinical and laboratory data at the time of PJP diagnostic workup*					
Length of hospital stay in days, median (IQR) (missing = 44)	8 (2–19)	3 (1–10)	9 (3–20)	0.98 (0.96–1.00)	0.013
Length of ICU stay in days, median (IQR) (missing = 19)	1 (0–6)	0 (0–1)	2 (0–8)	0.92 (0.88–0.96)	0.001
ARDS (missing = 31)	281 (53.2)	59 (57.8)	222 (52.1)	1.26 (0.82–1.95)	0.30
Invasive mechanical ventilation (missing = 11)	300 (54.7)	45 (40.2)	255 (58.5)	0.48 (0.31–0.73)	< 0.001
SOFA score, median (IQR) (missing = 4)	7 (4–10)	8 (4–10)	7 (4–9)	0.97 (0.92–1.02)	0.22
Septic shock (missing = 29)	159 (30.0)	29 (26.4)	130 (30.9)	0.80 (0.50–1.28)	0.35
CRRT (missing = 23)	61 (11.4)	8 (7.5)	53 (12.3)	0.58 (0.27–1.26)	0.17
CT scan performed (missing = 18)	299 (55.3)	69 (62.2)	230 (53.5)	1.43 (0.93–2.19)	0.10
No ground-glass opacities	23 (4.3)	0 (0)	23 (5.3)		§
Unilateral ground-glass opacities	7 (1.3)	3 (2.7)	4 (0.9)	2.95 (0.65–13.35)	0.16
Bilateral ground-glass opacities	68 (12.6)	22 (19.8)	46 (10.7)	2.05 (1.17–3.57)	0.012
Unilateral ground-glass and consolidations	20 (3.7)	1 (0.9)	19 (4.4)	0.20 (0.03–1.48)	0.11
Bilateral ground-glass and consolidations	175 (32.3)	39 (35.1)	136 (31.6)	1.16 (0.75–1.80)	0.50
Blood neutrophil count in cells × 10^–3^/mm^3^, median (IQR) (missing = 60)	7.3 (4.2–12.3)	6 (3.2–10.8)	7.7 (4.5–13.1)	0.96 (0.93–1.00)	0.03
Blood lymphocyte count in cells × 10^–3^/mm^3^, median (IQR) (missing = 115)	0.6 (0.3–1.1)	0.5 (0.2–0.9)	0.7 (0.4–1.2)	0.54 (0.37–0.79)	0.002
Serum CRP in mg/L, median (IQR) (missing = 35)	92 (23–186)	115.5 (42.3–217.5)	83 (20–179)	1.00 (1.00–1.00)	0.087
Serum PCT in ng/mL, median (IQR) (missing = 168)	0.9 (0.2–4.3)	0.4 (0.2–1.1)	1 (0.3–5.7)	1.0 (0.99–1.00)	0.25

AIDS: Acquired immune deficiency syndrome; ALL: Acute lymphocytic leukemia; AML: Acute myeloid leukemia; ARDS: Acute respiratory distress syndrome; CI: confidence interval;  COPD: Chronic obstructive pulmonary disease; CRRT: Continuous renal replacement therapy; CRP: C-reactive protein; CT: Computer tomography; HIV: Human immunodeficiency virus; HSCT: Hematopoietic stem cell transplantation; ICU: Intensive care unit; IQR: interquartile range; IVIG: Intravenous immunoglobulin; NYHA: New York Heart Association; OR: odds ratio; PCT: Procalcitonin; PJP: *Pneumocystis jirovecii* pneumonia; SOFA: Sequential Organ Failure Assessment

*Data reported as no. (%) unless otherwise indicated

^§^Logistic model not converging

**Table 3:** Multivariable analysis of factors associated with presumptive/proven PJP

Model A*	OR (95% CI)	*P*
AIDS	3.31 (1.13–9.64)	0.029^§^
Non-Hodgkin Lymphoma	3.71 (1.23–11.18)	0.020^§^
Vasculitis	5.95 (1.07–33.22)	0.042^§^
Metastatic solid tumor	4.31 (1.76–10.53)	0.001^§^
Invasive mechanical ventilation	0.43 (0.24–0.80)	0.007^§^
Blood lymphocyte count in cells × 10^–3^/mm^3^	0.64 (0.42–1.00)	0.049^§^
Serum CRP in mg/L	1.00 (1.00–1.01)	0.061
Bilateral ground glass	2.19 (1.01–4.78)	0.048^§^

Model B**	OR (95% CI)	*P*
AIDS	4.06 (1.24–13.28)	0.021^§^
Non-Hodgkin Lymphoma	3.42 (0.85–13.80)	0.084
Vasculitis	6.34 (1.09–36.76)	0.039^§^
Metastatic solid tumor	7.05 (2.45–20.31)	< 0.001^§^
Invasive mechanical ventilation	0.61 (0.29–1.28)	0.192
Blood lymphocyte count in cells × 10^–3^/mm^3^	0.442 (0.17–1.18)	0.102
Serum CRP in mg/L	1.16 (0.74–1.81)	0.512
Bilateral ground glass	2.89 (1.14–7.32)	0.025^§^

AIDS, acquired immune deficiency syndrome; CI, confidence interval; CRP, C-reactive protein; OR, odds ratio; PJP, *Pneumocystis jirovecii* pneumonia

*Only variable retained in the final multivariable model are presented in the table

**Generalized linear mixed model (GLMM) with center as random effect. Convergence of the mixed model was obtained after standardization of continuous variables and using the “bobyqa” optimizer. The model was built using the glmer function in the lme4 package for R Statistical Software (version 3.6.0, R Foundation for Statistical Computing, Vienna, Austria)

^§^*P* < 0.05

**Fig. 2:**
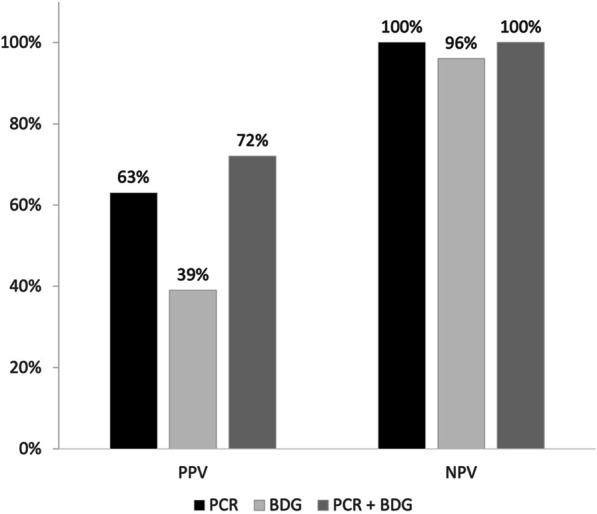
PPV and NPV for presumptive/proven PJP of serum BDG and respiratory *Pneumocystis* PCR both separately and in combination. BDG, (1,3)-β-D-glucan; NPV, negative predictive value; PCR, polymerase chain reaction; PJP, *Pneumocystis jirovecii* pneumonia; PPV, positive predictive value. Criteria for positivity used for the comparisons in the graph are manufactures’ cut-offs for BDG (80 pg/mL for the Fungitell assay and 11 pg/mL for the Wako assay) and any positive result for respiratory *Pneumocystis* PCR (see study methods). For the combination (PCR plus BDG), the reported PPV was obtained when both markers were concordant in indicating PJP (both positive), while the reported NPV was obtained when both markers when concordant in indicating no PJP (both negative)

The diagnostic performance of respiratory *Pneumocystis* PCR for the diagnosis of presumptive/proven PJP and for proven PJP only is shown in Table 4. Overall, respiratory *Pneumocystis* PCR was performed in 561/600 patients (93.5%), with results available for 550/561 patients (in the remaining 11 patients, *Pneumocystis* PCR was performed but the results were not provided). Using any positive results for the molecular detection of *Pneumocystis* DNA as the criterion for positivity, respiratory *Pneumocystis* PCR showed 100% sensitivity and 100% NPV both for diagnosis of presumptive/proven PJP and for proven PJP (in this latter case the analysis was conducted in the subgroup of patients who underwent microscopy, 190/550, 34.5%). Specificity was 85% and 61% for the diagnosis of presumptive/proven PJP and proven PJP, respectively. In subgroups according to the type of respiratory specimen (sputum, tracheal aspirate, or bronchoalveolar lavage fluid) specificity for the diagnosis of presumptive/proven PJP was lower for *Pneumocystis* PCR performed on sputum (68%) compared with deeper samples (85% for both tracheal aspirate and bronchoalveolar lavage fluid [BALF]), with *Pneumocystis* PCR on BALF showing overall the best diagnostic performance (Table 4). The diagnostic performance of serum BDG for the diagnosis of presumptive/proven PJP and for proven PJP only is shown in Table 5. Overall, serum BDG testing was performed in 327/600 patients (54.5%), mostly employing the Fungitell assay (293/327, 89.6%), whereas the Wako assay was employed only in 34/327 cases (10.4%), as shown in Additional file 1: Table S1. The distribution of BDG values according to presumptive/proven PJP diagnosis is shown in Additional file 1: Figure S1 (results reported for the Fungitell assay). Based on Youden index, the best compromise between sensitivity and specificity for the diagnosis of presumptive/proven PJP using the Fungitell assay was a cut-off level of 230 pg/mL, showing 82% sensitivity and 82% specificity (figure S2). The diagnostic performance of respiratory *Pneumocystis* PCR and serum BDG in subgroups according to baseline disease/condition is available in Additional file 1: Tables S3 and S4, respectively. Although with moderate strength, correlations were identified between increasing serum BDG values and decreasing Ct values of *Pneumocystis* PCR on BALF (63 pairs, *p* < 0.001, Spearman r − 0.49) and between increasing serum BDG values and decreasing Ct values of *Pneumocystis* PCR on tracheal aspirate (10 pairs, *p* = 0.041, Spearman r -0.67). Finally, an increased PPV was observed when both respiratory *Pneumocystis* PCR and serum BDG were positive in comparison with the performances of the two markers separately, whereas when both were negative their NPV did not substantially vary compared with the NPV obtained using only respiratory *Pneumocystis* PCR or only serum BDG (Fig. 2 and Additional file 1: Table S5).

**Table 4:** Performance of respiratory *Pneumocystis* PCR for the diagnosis of PJP*

Population	PJP (TP/total)	No PJP (TN/total)	Sensitivity % (95% CI)	Specificity % (95% CI)	PPV % (95% CI)	NPV % (95% CI)	LR + (95% CI)	LR- (95% CI)
*Diagnosis of presumptive/proven PJP*								
All respiratory *Pneumocystis* PCR**	111/111	373/439	100 (97–100)	85 (81–88)	63 (55–70)	100 (99–100)	6.7 (5.3–8.3)	0.0 §
Sputum *Pneumocystis* PCR***	10/10	19/28	100 (69–100)	68 (48–84)	53 (29–76)	100 (82–100)	3.1 (1.8–5.3)	0.0 §
Tracheal aspirate *Pneumocystis* PCR***	10/10	63/74	100 (69–100)	85 (75–92)	48 (26–70)	100 (94–100)	6.7 (3.9–11.6)	0.0 §
BALF *Pneumocystis* PCR***	95/95	299/351	100 (96–100)	85 (81–89)	65 (56–72)	100 (99–100)	6.8 (5.3–8.7)	0.0 §
Diagnosis of proven PJP****								
*Pneumocystis* PCR vs. microscopy (ref) *****	27/27	100/163	100 (87–100)	61 (53–69)	30 (21–41)	100 (96–100)	2.6 (2.1–3.1)	0.0 §

BALF, bronchoalveolar lavage fluid; CI, confidence interval; LR−, negative likelihood ratio; LR+, positive likelihood ratio; NPV, negative predictive value; PCR, polymerase chain reaction; PJP, *Pneumocystis jirovecii* pneumonia; PPV, positive predictive value; TN, true negative; TP, true positive

*Quantitative or qualitative *Pneumocystis* PCR (according to locally implemented laboratory developed tests or commercial assays, for details see Additional file 1: Table S1) on respiratory specimens (sputum, tracheal aspirate, and/or bronchoalveolar lavage fluid). Patients with neither a “PJP” nor a “no PJP” diagnosis (i.e., “diagnosis inconclusive”, see study methods) were conservatively classified as “no PJP” to reduce overestimation of the diagnostic performance of *Pneumocystis* PCR (a higher frequency of DNA detection was indeed registered in patients with inconclusive diagnosis than in the entire “no PJP” population)

**The criterion for PCR positivity was defined as the detection of *Pneumocystis* DNA in any respiratory specimen (sputum, tracheal aspirate, and/or bronchoalveolar lavage fluid). For reference definitions of presumptive and proven PJP see methods

***Not mutually exclusive since some patients underwent *Pneumocystis* PCR testing on different types of respiratory samples (sputum, tracheal aspirate, and/or BALF)

****Evaluated in the subgroup of patients tested for *Pneumocystis* microscopy (Crystal Violet, May-Grünwald-Giemsa, Wright-Giemsa, Rapid Giemsa-like stains, Direct Fluorescent Antibody, Methenamine Silver, or Toluidine Blue O according to local procedures) on respiratory specimens

*****Positivity of microscopy as reference was defined as at least one positive tested sample/s (sputum, tracheal aspirate, and/or bronchoalveolar lavage fluid)

^§^No false negatives in the tested sample (consider the presence of incorporation bias, see methods)

**Table 5:** Performance of serum BDG for the diagnosis of PJP*

Population	PJP (TP/total)	No PJP (TN/total)	Sensitivity % (95% CI)	Specificity % (95% CI)	PPV % (95% CI)	NPV % (95% CI)	LR + (95% CI)	LR- (95% CI)
*Diagnosis of presumptive/proven PJP*								
All patients with serum BDG testing**	49/57	192/270	86 (74–94)	71 (65–76)	39 (30–48)	96 (92–98)	3.0 (2.4–3.7)	0.2 (0.1–0.4)
Fungitell test	46/51	169/242	90 (79–97)	70 (64–76)	39 (30–48)	97 (93–99)	3.0 (2.4–3.7)	0.1 (0.1–0.3)
Wako test	3/6	23/28	50 (12–88)	82 (63–94)	38 (9–76)	88 (70–98)	2.8 (0.9–8.7)	0.6 (0.3–1.4)
Diagnosis of proven PJP***								
Serum BDG versus microscopy (ref) ****	14/19	58/95	74 (49–91)	61 (51–71)	27 (16–42)	92 (82–97)	1.9 (1.3–2.7)	0.4 (0.2–0.9)

BDG, (1,3)-ß-D-glucan; CI, confidence interval; LR-, negative likelihood ratio; LR + , positive likelihood ratio; NPV, negative predictive value; PJP, *Pneumocystis jirovecii* pneumonia; PPV, positive predictive value; TN, true negative; TP, true positive

*Patients with neither a “PJP” nor a “no PJP” diagnosis (i.e., “diagnosis inconclusive”, see study methods) were conservatively classified as “no PJP” to reduce overestimation of the diagnostic performance of serum BDG (a higher frequency of positive serum BDG was indeed registered in patients with inconclusive diagnosis than in the entire “no PJP” population)

**A serum BDG value equal or above the manufacturer cut-off (80 pg/mL and 11 pg/mL for the Fungitell assay and Wako assay, respectively) was defined as the criterion for serum BDG positivity. For reference definitions of presumptive and proven PJP see methods

***Evaluated in the subgroup of patients tested for *Pneumocystis* microscopy (Crystal Violet, May-Grünwald-Giemsa, Wright-Giemsa, Rapid Giemsa-like stains, Direct Fluorescent Antibody, Methenamine Silver, or Toluidine Blue O according to local procedures) on respiratory specimens

****Positivity of microscopy as reference was defined as at least one positive tested sample/s (sputum, tracheal aspirate, and/or bronchoalveolar lavage fluid)

Overall, 98.3% of patients with presumptive/proven PJP received PJP therapy (113/115, 98.3%), mostly with trimethoprim/sulfamethoxazole (96.5%, 109/113). Information on concomitant/alternative infections due to pathogens other than *Pneumocystis jirovecii* are available in Additional file 1: Table S6. As shown in Additional file 1: figures S3 and S4, both cumulative 30-day mortality and cumulative 90-day mortality were high and similar in patients with and without PJP (i.e., with other causes of pneumonia, see Additional file 1: Table S6) in our study population. More in detail, cumulative 30-day mortality was 52% and 47% in patients with presumptive/proven PJP and without PJP, respectively (log-rank test, *p* = 0.74), and cumulative 90-day mortality was 67% and 68% in patients with presumptive/proven PJP and without PJP, respectively (log-rank test, *p* = 0.76). Cumulative 30-day and cumulative 90-day mortality stratified by baseline condition/disease (HIV infection vs. other baseline diseases or conditions) in patients with presumptive/proven PJP are also available in Additional file 1: Figures S5 and S6.

## Discussion

In the present large, multinational cohort study, we showed that: (*i*) more patients admitted to ICU are subjected to PJP diagnostic workup compared with the past, with a reduction in the relative frequency of patients with HIV infection and an increase in patients with hematological and neoplastic diseases, solid organ transplant, and inflammatory diseases; (*ii*) combining serum BDG and respiratory *Pneumocystis* PCR improved the PPV for the diagnosis of PJP in critically ill patients admitted to ICU.

Although patients with HIV infection and AIDS were still those eventually showing a higher frequency of PJP in our cohort (55.9%), the absolute number of HIV patients with PJP was much lower than the absolute number of patients with PJP and other predisposing diseases or conditions, which is in line with results from previous smaller cohorts [5, 6]. Overall, while HIV infection remains one of the factors conferring the highest risk of PJP, the expanding denominator of ICU patients with other predisposing conditions overall makes PJP more commonly encountered in non-HIV critically ill patients nowadays, a fact that should be considered by ICU physicians caring for patients with pneumonia and forms of immunosuppression other than HIV infection. Besides AIDS, other predisposing conditions showing an independent association with PJP in multivariable models were non-Hodgkin lymphoma, metastatic solid tumor, and vasculitis. Among factors inversely associated with PJP diagnosis, the apparently reduced risk conferred by invasive mechanical ventilation at the time of PJP diagnostic workup may reflect the fact that PJP was most frequently the reason for ICU admission and for requirement of invasive mechanical ventilation rather than a complication developing during ICU stay. The increased risk conferred by low lymphocyte counts is conversely more likely testifying a true risk factor, in line with what observed in HIV patients with low CD4 + lymphocyte cell count [21]. Of note, although a few associations did not retain statistical significance in the additional multivariable model including center as a random effect (model B), the directions of effects were in line with those observed in the primary model (model A), suggesting limited influence of between-center variability on results. This is important considering the possible heterogeneity in the diagnostic workup for PJP in the different participating centers (e.g., testing in all patients with interstitial pneumonia vs. testing only in patients with risk factors), and strengthens the generalizability of the detected associations.

Regarding the secondary objective of assessing the diagnostic performance for PJP of respiratory *Pneumocystis* PCR and serum BDG, in our study respiratory *Pneumocystis* PCR showed high sensitivity (100%) and relatively low specificity (up to 85% for BALF, and lower for sputum samples) for the diagnosis of presumptive/proven PJP. Notably, although the majority of patients in our cohort underwent PCR testing on BALF, some did not; this could represent an important limitation for the diagnostic assessment of PJP in real life setting, considering the overall better diagnostic performance of BALF testing compared with other respiratory samples [22–25]. In our opinion, some important considerations may stem from our results: (i) in presence of a negative respiratory *Pneumocystis* PCR, PJP seems to be very unlikely (i.e., PCR retains a high NPV), even in presence of a positive serum BDG; (ii) concomitant positivity of respiratory *Pneumocystis* PCR and serum BDG increases PPV in comparison with PCR alone, potentially helping to distinguish between *Pneumocystis* colonization and infection in the absence of established cut-offs for quantitative molecular tests (and/or when qualitative molecular tests are employed) [25, 26].

A first important limitation, related to our primary descriptive analysis, is the lack of precise information about previous/ongoing corticosteroid treatment (e.g., dosage, length of therapy), a recognized predisposing condition to PJP, that was unfortunately unavailable retrospectively. However, in our opinion, this limitation eventually did not undermine the identification of the spectrum of baseline diseases nowadays encountered in critically ill patients with PJP. Future studies with a more detailed information on steroid treatments could help to define the possible reasons for the low prevalence of patients on PJP prophylaxis in our cohort despite the high frequency of patients with at least one predisposing disease/condition. Notably, a low frequency of PJP prophylaxis in non-HIV patients at risk of PJP was already reported by Dunbar and colleagues and by Roux and colleagues [27, 28]. The lack of detailed information about corticosteroid treatments also precluded the evaluation of the diagnostic performance of respiratory *Pneumocystis* PCR and serum BDG using the EORTC/MSGERC definition of probable PJP, although it should be highlighted that this would have resulted in a significant incorporation bias [14]. Of course, our definition of presumptive PJP, although more suitable for the aims of the present study, is not standardized and also still hampered by a nonnegligible risk of incorporation bias, that should be necessarily acknowledged. This also considering the relatively low frequency of patients who underwent computerized tomography (56.5%), the absence of which could have further hampered the identification of PJP and its differential diagnosis in some cases. Second, in the assessment of the diagnostic performance of respiratory *Pneumocystis* PCR we defined any positive result as the criterion for positivity, to preserve homogeneity of interpretation despite the wide heterogeneity of employed molecular tests across centers (Additional file 1: Table S1), whereas higher fungal loads are typically more indicative of active infection than low fungal loads [29]. In this regard, the highly fragmented data in terms of Ct values available for this study prevented us from reliably assessing potential thresholds to distinguish between colonization and infection. A third limitation arises from the recognized suboptimal sensitivity of the diagnostic gold standard (microscopy) [30] and from the low number of microscopy tests performed in our real life cohort, that forced us to strongly rely on a definition of presumptive disease as diagnostic reference. Finally, it should be noted that mortality rates reported in this study, either for PJP or for pneumonia caused by other pathogens, are only presented descriptively and unadjusted for other prognostic factors.

In conclusion, PJP in critically ill patients admitted to ICU is nowadays most encountered in non-HIV patients. Our results also suggest a high NPV of respiratory *Pneumocystis* PCR and that combining PCR with serum BDG increases the otherwise modest PPV, although further dedicated study remains necessary to precisely delineate their role within PJP diagnostic algorithms in the ICU.

### Supplementary Information

**Additional file 1.** Supplementary tables S1-S6 and supplemtary figures S1-S6.

## Data Availability

The data presented in this study will be available from the corresponding author on reasonable request and provided all regulatory and privacy requirements are fulfilled.
