# Supplementary material for: Pneumocystis jirovecii pneumonia in intensive care units: a multicenter study by ESGCIP and EFISG
Source: Crit Care. 2023 Aug 24;27:323. doi: 10.1186/s13054-023-04608-1 (PMC10464114; doi:10.1186/s13054-023-04608-1)
Supplement: Supplementary file 1 — Additional file 1. Supplementary tables S1-S6 and supplemtary figures S1-S6. [file 13054_2023_4608_MOESM1_ESM.pdf]

Supplementary Table S1. Overview of conventional and molecular assays employed by the study centers for the diagnosis of PJP.

Diagnostic test** (N)		n (%)	Methods (no. of employing centers)							
**Microscopy (234)**			CV (2)	DFA (6)	MGG (3)	MS (3)	PAP (1)	RGS (1)	TBO (2)	WG (1)
	Sputum	22 (10)	2	12	1	-	-	8	1	-
	Tracheal aspirate	50 (21)	1	43	2	1	1	2	-	-
	BALF	162 (69)	7	60	33	7	2	24	27	1
**Real-time PCR* (588)**			Commercial (9)				LDT (11)			
	Sputum	39 (7)	15				24			
	Tracheal aspirate	84 (14)	66				18			
	BALF	451 (77)	282				169			
	*Blood****	14 (2)	13				1			
**BDG (327)**			Fungitell Assay (11)				Wako β-Glucan (2)			
	Serum	327 (100)	293				34			

Supplementary table S1 legend. BDG, (1,3)-ß-D-glucan; CV, Crystal Violet; DFA, direct fluorescent antibody; MGG, May-Grünwald-Giemsa; MS, Methenamine Silver (e.g., Grocott-Gomori); PAP, Papanicolaou; RGS, Rapid Giemsa-like stains (e.g,Diff-Quik, Hema-Quik); TBO, Toluidine Blue O; WG, Wright-Giemsa; BALF, bronchoalveolar lavage fluid; LDT: laboratory developed test. *Quantitative or qualitative *Pneumocystis* PCR (according to locally implemented assays). ** Not mutually exclusive; furthermore, both microscopy and PCR were sometimes performed on more than one type of respiratory sample from the same patient. *** Patients who underwent blood PCR also underwent respiratory PCR and were classified based on the results of respiratory PCR.

Supplementary Table S2. Baseline characteristics of patients with proven and presumptive PJP

Variable*	Patients with proven/presumptive PJP n = 115 (100)	Patients with proven PJP n = 31 (27.0)	Patients with presumptive PJP n = 84 (73.0)
**Demographic**			
Age in years, median (IQR)	60 (49 – 70)	61 (47 – 70)	60 (49 – 68)
Female sex	35 (30.4)	9 (29.0)	26 (30.9)
**Medical history**			
HIV infection (missing = 2)	22 (19.5)	9 (29.0)	13 (15.5)
AIDS (missing = 1)	19 (17.0)	7 (22.6)	12 (14.3)
Solid organ transplant	6 (5.2)	2 (6.4)	4 (4.8)
Liver	0 (0)	0 (0)	0 (0)
Kidney	5 (4.3)	2 (6.4)	3 (3.6)
Lung	1 (0.9)	0 (0)	1 (1.2)
Heart	0 (0)	0 (0)	0 (0)
Other	0 (0)	0 (0)	0 (0)
Hematological malignancy (missing = 3)	36 (31.3)	9 (29.0)	27 (32.1)
AML	4 (3.4)	1 (3.2)	3 (3.6)
ALL	3 (2.6)	1 (3.2)	2 (2.4)
Hodgkin lymphoma	2 (1.7)	0 (0)	2 (2.4)
Non-Hodgkin lymphoma	16 (13.9)	4 (12.9)	12 (14.3)
Other	8 (6.9)	2 (6.4)	6 (7.1)
HSCT	12 (10.4)	1 (3.2)	11 (13.1)
Inflammatory disease	17 (14.8)	2 (6.4)	15 (17.9)
Rheumatoid Arthritis	3 (2.6)	0 (0)	3 (3.6)
Systemic Lupus Erythematosus	1 (0.9)	0 (0)	1 (1.2)

Polymyositis-Dermatomyositis	0 (0)	0 (0)	0 (0)
Inflammatory bowel disease	2 (1.7)	0 (0)	2 (2.4)
Scleroderma	0 (0)	0 (0)	0 (0)
Vasculitis	4 (3.4)	0 (0)	4 (4.8)
Mixed Connective Tissue Disease	1 (0.9)	1 (3.2)	0 (0)
Autoimmune hepatitis	1 (0.9)	0 (0)	1 (1.2)
Sarcoidosis	1 (0.9)	0 (0)	1 (1.2)
Autoimmune hemolytic anemia	1 (0.9)	0 (0)	1 (1.2)
Myasthenia gravis	0 (0)	0 (0)	0 (0)
Other	3 (2.6)	1 (3.2)	2 (2.4)
Solid tumor (missing = 1)	28 (24.6)	7 (22.6)	21 (25.0)
Metastatic solid tumor (missing = 3)	14 (12.6)	4 (12.9)	10 (11.9)
COPD (missing = 1)	11 (9.6)	4 (12.9)	7 (8.3)
Chronic pulmonary diseases other than COPD (missing = 2)	9 (8.0)	0 (0)	9 (10.7)
Asthma	2 (1.8)	0 (0)	2 (2.4)
Cystic fibrosis	1 (0.9)	0 (0)	1 (1.2)
Interstitial lung disease/pulmonary fibrosis	2 (1.8)	0 (0)	2 (2.4)
Other	4 (3.5)	0 (0)	4 (4.8)
Chronic kidney disease (missing = 1)	13 (11.4)	3 (9.7)	10 (11.9)
Chronic liver disease (missing = 1)	9 (7.9)	2 (6.4)	7 (8.3)
NYHA score >2 (missing = 23)	13 (14.1)	2 (6.4)	11 (13.1)
Age-adjusted Charlson score, median (IQR)	4 (3 – 7)	6 (4 – 8)	4 (2 – 6.3)
Previous major surgery (within 30 days) (missing = 4)	6 (5.4)	1 (3.2)	5 (5.9)
Previous chemotherapy (within 30 days) (missing = 2)	39 (34.5)	12 (38.7)	27 (32.1)
Previous radiotherapy (within 30 days) (missing = 1)	10 (8.8)	3 (9.7)	7 (8.3)
Previous IVIG therapy (within 30 days) (missing = 8)	8 (7.5)	1 (3.2)	7 (8.3)

Previous albumin therapy (within 30 days) (missing = 9)	7 (6.6)	0 (0)	7 (8.3)
Previous blood transfusions (within 30 days) (missing = 10)	21 (20.0)	7 (22.6)	14 (16.7)
PJP prophylaxis (missing data = 25)	1 (1.1)	0 (0)	1 (1.2)
**Clinical and laboratory data at the time of PJP diagnostic workup**			
Length of hospital stay in days, median (IQR) (missing = 25)	3 (1 – 10)	5 (1.3 – 12.7)	2 (0.7 – 9)
Length of ICU stay in days, median (IQR)	0 (-1 – 1)	0 (-0.5 – 1)	0 (-1 – 1)
ARDS (missing = 13)	59 (57.8)	13 (41.9)	46 (54.8)
Invasive mechanical ventilation (missing = 3)	45 (40.2)	10 (32.3)	35 (41.7)
SOFA score, median (IQR) (missing = 4)	8 (4 – 10)	7 (4 – 10)	5 (3.7 – 9)
Septic shock (missing = 5)	29 (29.0)	10 (32.3)	19 (22.6)
CRRT (missing = 9)	8 (7.5)	2 (3.2)	6 (7.1)
CT scan performed (missing = 4)	69 (62.2)	22 (70.9)	47 (55.9)
No ground-glass opacities	0 (0)	0 (0)	0 (0)
Unilateral ground-glass opacities	3 (2.7)	0 (0)	3 (3.6)
Bilateral ground-glass opacities	22 (19.8)	5 (16.1)	17 (20.2)
Unilateral ground-glass and consolidations	1 (0.9)	0 (0)	1 (1.2)
Bilateral ground-glass and consolidations	39 (35.1)	17 (54.8)	22 (26.2)
Blood neutrophil count in cells × $10^{-3}/mm^3$, median (IQR) (missing = 13)	6 (3.2 – 10.8)	5.2 (2.7 – 6.3)	6.7 (3.5 – 10.9)
Blood lymphocyte count in cells × $10^{-3}/mm^3$, median (IQR) (missing = 14)	0.5 (0.2 – 0.9)	0.5 (0.1 – 0.7)	0.5 (0.2 – 0.9)
Serum CRP in mg/L, median (IQR) (missing = 7)	115.5 (42.3 – 217.5)	77.1 (35 – 164)	125 (46 – 220)
Serum PCT in ng/mL, median (IQR) (missing = 51)	0.4 (0.2 – 1.1)	0.7 (0.2 – 1.1)	0.4 (0.2 – 2.6)

Supplementary table S2 legend. AIDS: Acquired immune deficiency syndrome; ALL: Acute lymphocytic leukemia; AML: Acute myeloid leukemia; ARDS: Acute respiratory distress

syndrome; COPD: Chronic obstructive pulmonary disease; CRRT: Continuous renal replacement therapy; CRP: C-reactive protein; CT: Computer tomography; HIV: Human immunodeficiency virus; HSCT: Hematopoietic stem cell transplantation; ICU: Intensive care unit; IQR: interquartile range; IVIG: Intravenous immunoglobulin; NYHA: New York Heart Association; PCT: Procalcitonin; PJP: *Pneumocystis jirovecii* pneumonia; SOFA: Sequential Organ Failure Assessment.
* Data reported as no. (%) unless otherwise indicated. Number of missing values, impacting denominator and frequency calculation, are reported in parenthesis for each variable.

Supplementary Table S3. Performance of *Pneumocystis* PCR on respiratory specimens for the diagnosis of presumptive/proven PJP in subgroups by baseline/concomitant condition/disease*

Baseline/concomitant condition/disease	PJP (TP/total)	No PJP (TN/total)	Sensitivity % (95% CI)	Specificity % (95% CI)	PPV % (95% CI)	NPV % (95% CI)	LR+ (95% CI)	LR- (95% CI)
HIV infection	22/22	20/26	100 (85-100)	77 (56-91)	79 (59-92)	100 (83-100)	4.3 (2.2, 8.7)	0.0 §
AIDS	19/19	10/14	100 (82-100)	71 (42-92)	83 (61-95)	100 (69-100)	3.5 (1.5-8.0)	0.0 §
Haematological malignancy	34/34	77/93	100 (90-100)	83 (74-90)	68 (53-80)	100 (95-100)	5.8 (3.7-9.1)	0.0 §
Solid organ transplant	6/6	46/53	100 (54-100)	87 (75-95)	46 (19-75)	100 (92-100)	7.6 (3.8- 15.1)	0.0 §
Inflammatory disease	17/17	54/70	100 (80-100)	77 (66-86)	52 (34-69)	100 (93-100)	4.4 (2.8-6.7)	0.0 §
COVID-19 pneumonia	5/5	60/66	100 (48-100)	91 (81-97)	45 (17-77)	100 (94-100)	11.0 (5.1- 23.6)	0.0 §

Supplementary table S3 legend. AIDS, acquired immune deficiency syndrome; CI, confidence interval; COVID-19, coronavirus disease 2019; LR-, negative likelihood ratio; LR+, positive likelihood ratio; NPV, negative predictive value; PCR, polymerase chain reaction; PJP, *Pneumocystis jirovecii* pneumonia; PPV, positive predictive value; TN, true negative; TP, true positive.

* Quantitative or qualitative *Pneumocystis* PCR (according to locally implemented laboratory developed tests or commercial assays, for detail see supplementary table S1) on respiratory specimens (sputum, tracheal aspirate, and/or bronchoalveolar lavage fluid). The criterion for PCR positivity was defined as detection of any amount of *Pneumocystis* DNA on at least one respiratory specimen. For the reference definitions of presumptive and proven PJP see study methods. Patients with neither a “PJP” nor a “no PJP” diagnosis (i.e., “diagnosis inconclusive”, see study methods) were conservatively classified as “no PJP” to reduce overestimation of the diagnostic performance of *Pneumocystis* PCR (a higher frequency of DNA detection was indeed registered in patients with inconclusive diagnosis than in the entire “no PJP” population).

§ No false negative cases in the tested sample

Supplementary Table S4. Performance of serum BDG for the diagnosis of presumptive/proven PJP in subgroups by baseline/concomitant condition/disease*

Baseline/concomitant condition/disease	PJP (TP/total)	No PJP (TN/total)	Sensitivity % (95% CI)	Specificity % (95% CI)	PPV % (95% CI)	NPV % (95% CI)	LR+ (95% CI)	LR- (95% CI)
HIV infection	6/7	8/13	86 (42-100)	62 (32-86)	55 (23-83)	89 (52-100)	2.2 (1.1-4.7)	0.2 (0.0- 1.5)
AIDS	5/6	4/6	83 (36-100)	67 (22-96)	71 (29-96)	80 (28-99)	2.5 (0.8-8.2)	0.3 (0.0- 1.6)
Haematological malignancy	16/17	47/63	94 (71-100)	75 (62-85)	50 (32-68)	98 (89-100)	3.7 (2.4-5.8)	0.1 (0.0- 0.5)
Solid organ transplant	4/4	17/26	100 (40-100)	65 (44-83)	31 (9-61)	100 (80-100)	2.9 (1.7-4.9)	0.0 §
Inflammatory disease	7/9	30/48	78 (40-97)	62 (47-76)	28 (12-49)	94 (79-99)	2.1 (1.3-3.4)	0.4 (0.1- 1.2)
COVID-19 pneumonia	0/2	39/47	0 (0-84) §§	83 (69-92)	0 (0-37) §§	95 (83-99)	0.0 §§	1.2 (1.1- 1.4)

Supplementary table S4 legend. AIDS, acquired immune deficiency syndrome; BDG, (1,3)-ß-D-glucan; CI, confidence interval; COVID-19, coronavirus disease 2019; HIV, human immunodeficiency virus; LR-, negative likelihood ratio; LR+, positive likelihood ratio; NPV, negative predictive value; PJP, *Pneumocystis jirovecii* pneumonia; PPV, positive predictive value; TN, true negative; TP, true positive.

* A serum BDG value equal or above the manufacturer cut-off (80 pg/mL and 11 pg/mL for the Fungitell assay and Wako assay, respectively) was defined as the criterion for serum BDG positivity. For reference definitions of presumptive and proven PJP see methods. Patients with neither a “PJP” nor a “no PJP” diagnosis (i.e., “diagnosis inconclusive”, see study methods) were conservatively classified as “no PJP” to reduce overestimation of the diagnostic performance of serum BDG (a higher frequency of positive serum BDG was indeed registered in patients with inconclusive diagnosis than in the entire “no PJP” population).

§ No false negatives in the tested sample

§§ No true positives in the tested sample (only two cases of PJP were found in COVID-19 patients who underwent serum BDG testing)

Supplementary table S5. Performance of respiratory *Pneumocystis* PCR combined with serum BDG for the diagnosis of presumptive/proven PJP*

Population	PJP (TP/total)	No PJP (TN/total)	Sensitivity % (95% CI)	Specificity % (95% CI)	PPV % (95% CI)	NPV % (95% CI)	LR+ (95% CI)	LR- (95% CI)
Positive PCR and/or positive BDG	55/55	186/236	100 (94-100)	79 (73-84)	52 (42-62)	100 (98-100)	4.7 (3.7-6.0)	0.0 §
Positive PCR and positive BDG	47/55	218/236	85 (73-94)	92 (88-95)	72 (60-83)	96 (93-98)	11.2 (7.1-17.7)	0.2 (0.1-0.3)

Supplementary table S5 legend. BDG, (1,3)-ß-D-glucan; CI, confidence interval; LR-, negative likelihood ratio; LR+, positive likelihood ratio; NPV, negative predictive value; PCR, polymerase chain reaction; PJP, *Pneumocystis jirovecii* pneumonia; PPV, positive predictive value; TN, true negative; TP, true positive.

* The criterion for PCR positivity was defined as detection of any amount of *Pneumocystis* DNA on at least one respiratory specimen. A serum BDG value equal or above the manufacturer cut-off (80 pg/mL and 11 pg/mL for the Fungitell assay and Wako assay, respectively) was defined as the criterion for serum BDG positivity. For reference definitions of presumptive and proven PJP see methods. Patients with neither a "PJP" nor a "no PJP" diagnosis (i.e., "diagnosis inconclusive", see study methods) were conservatively classified as "no PJP" to reduce overestimation of the diagnostic performance of both PCR and BDG

*** Evaluated in the subgroup of patients tested for *Pneumocystis* microscopy (Crystal Violet, May-Grünwald-Giemsa, Wright-Giemsa, Rapid Giemsa-like stains, Direct Fluorescent Antibody, Methenamine Silver, or Toluidine Blue O according to local procedures) on respiratory specimens.

**** Positivity of microscopy as reference was defined as at least one positive tested sample/s (sputum, tracheal aspirate, and/or bronchoalveolar lavage fluid).

§ No false negatives in the tested sample.

Supplementary Table S6. Infections other than PJP in the study population

	All patients<br>n = 600 (100)	Patients with PJP*<br>n = 115 (19.2)	Patients without PJP<br>n = 444 (74.0)	PJP diagnosis inconclusive<br>n = 41 (6.8)
**Infection other than PJP****				
COVID-19 pneumonia (missing =3)	75 (12.6)	5 (4.4)	66 (14.9)	4 (9.8)
Bacterial pneumonia (missing = 11)	307 (52.1)	41 (36.6)	244 (55.8)	22 (55.0)
Invasive pulmonary aspergillosis (missing = 11)	33 (5.6)	6 (5.4)	25 (5.7)	2 (5.0)
Influenza (missing = 44)	42 (7.6)	1 (0.9)	38 (9.4)	3 (7.3)
CMV reactivation (missing = 44)	50 (9.0)	24 (22.0)	25 (6.2)	1 (2.4)
HSV reactivation (missing = 44)	22 (3.9)	13 (11.9)	7 (1.7)	2 (4.9)
Bacteremia (missing = 11)	131 (22.2)	29 (25.2)	96 (22.1)	6 (15.4)
Candidemia (missing = 5)	23 (3.9)	5 (4.4)	17 (3.9)	1 (2.4)

Supplementary table S6 legend. CI, confidence interval; CMV, cytomegalovirus; COVID-19, coronavirus disease 2019, HSV, herpesvirus; PJP, *Pneumocystis jirovecii* pneumonia.
* According to presumptive/proven PJP diagnosis (see study methods)
** reported as no. (%)

Supplementary Figure S1. Distribution of serum BDG levels in critically ill patients according to the diagnosis of presumptive/proven PJP

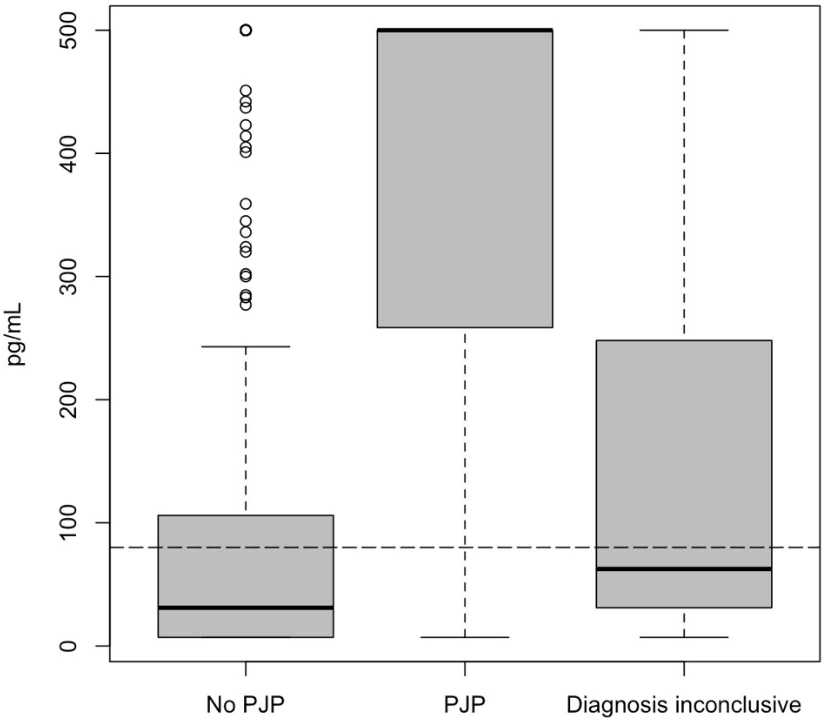

Supplementary figure S1 legend. Serum (1,3)-ß-D-glucan levels (BDG) expressed in pg/mL in critically ill patients tested with the Fungitell assay (n = 293) and divided into groups according to the presence of presumptive/proven *Pneumocystis jirovecii* pneumonia (PJP) diagnosis. The horizontal dotted line represents the cut-off for positivity (80 pg/mL), the thick bars represent the median values, the gray areas above and below the bars represent the upper and the lower quartiles, respectively. Values <7 pg/mL and >500 pg/mL were approximated and reported as 7 pg/mL and 500 pg/mL, respectively.

Supplementary Figure S2. ROC curve of the performance of serum BDG for the diagnosis of presumptive/proven PJP

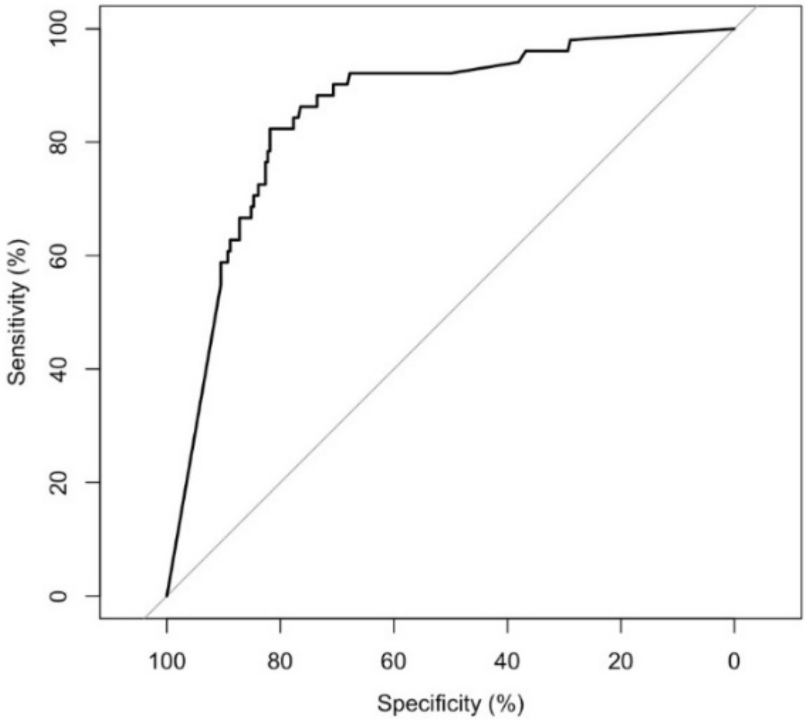

Supplementary figure S2 legend. Receiver operating characteristic (ROC) curve of the performance of serum (1,3)-ß-D-glucan (BDG) for the diagnosis of presumptive/proven PJP in critically ill patients tested with the Fungitell assay (n = 293). The area under the ROC curve was 0.854 (95% CI 0.799–0.962) and the point on the curve with the maximum Youden Index was 230 pg/mL (82% sensitivity and 82% specificity).

Supplementary Figure S3. Survival at 30 days in critically ill patients with and without presumptive/proven PJP

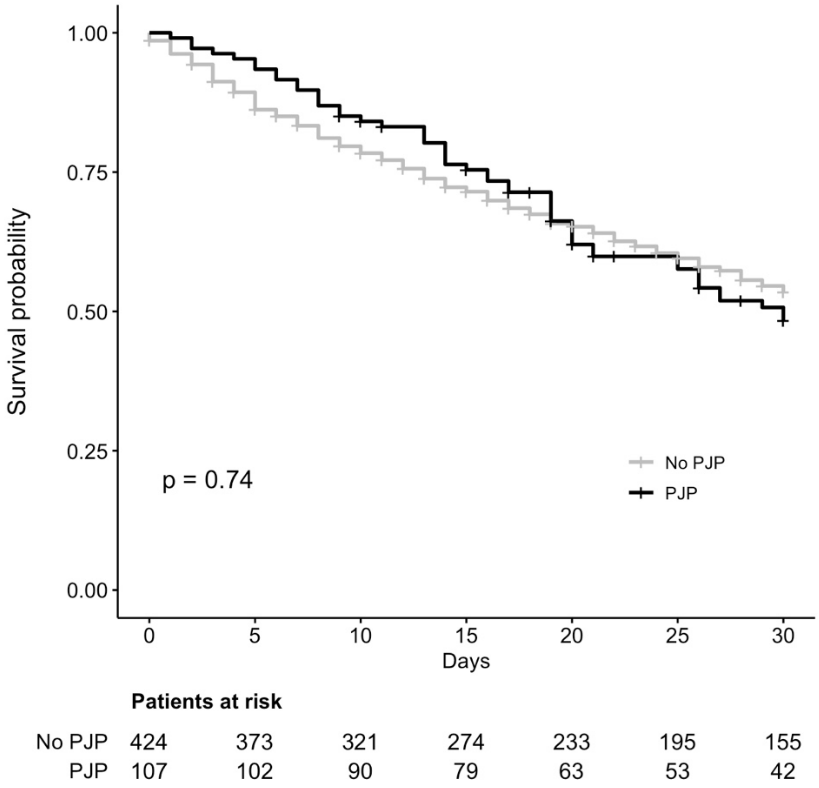

Supplementary figure S3 legend. Survival at 30 days in critically ill patients with and without presumptive/proven *Pneumocystis jirovecii* pneumonia (PJP). Follow-up data were available for 424/444 patients without PJP (95%) and 107/115 patients with presumptive/proven PJP (93%). The time of origin was set at the time of PJP diagnostic workup. Right-censoring was applied at the time of hospital discharge or at 30 days after the time of PJP diagnostic workup, whichever came first. The reported p value is from log-rank test. No difference in survival was also observed when including patients with inconclusive diagnosis of PJP (n = 41) either in the PJP or in the no PJP group (p = 0.70 and p = 0.76, respectively; curves not shown).

Supplementary Figure S4. Survival at 90 days in critically ill patients with and without presumptive/proven PJP

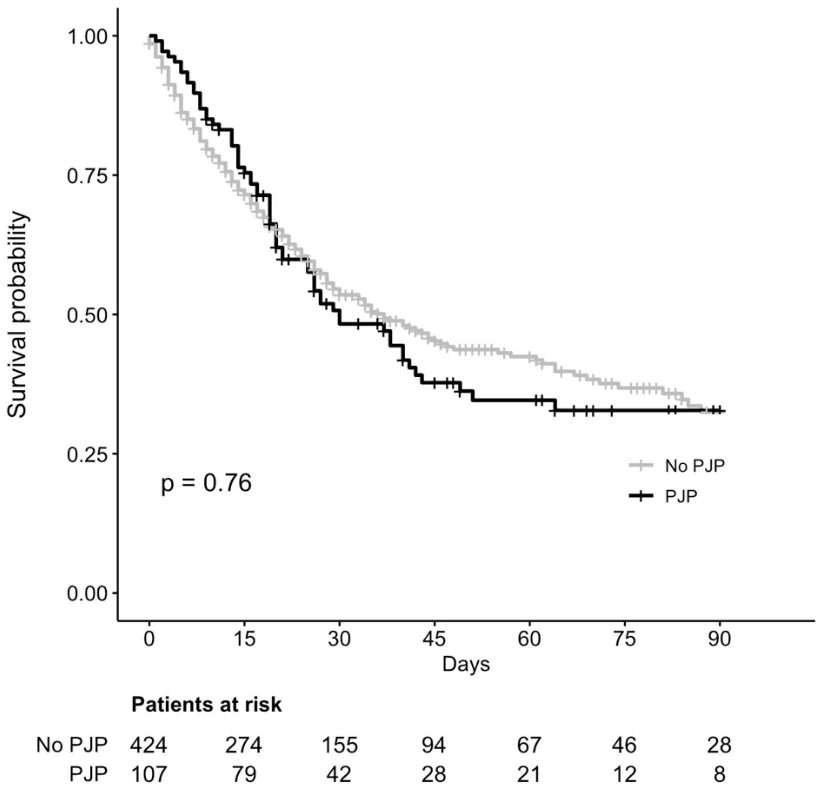

Supplementary figure S4 legend. Survival at 90 days in critically ill patients with and without presumptive/proven *Pneumocystis jirovecii* pneumonia (PJP) diagnosis. Follow-up data were available for 424/444 patients without PJP (95%) and 107/115 patients with presumptive/proven PJP (93%). The time of origin was set at the time of PJP diagnostic workup. Right-censoring was applied at the time of hospital discharge or at 90 days after the time of PJP diagnostic workup, whichever came first. The reported p value is from log-rank test. No difference in survival was also observed when including patients with inconclusive diagnosis of PJP (n = 41) either in the PJP or in the no PJP group (p = 0.93 and p = 0.71, respectively; curves not shown).

Supplementary Figure S5. Survival at 30 days in critically ill patients with PJP, stratified according to the presence of HIV infection vs. other baseline diseases/conditions

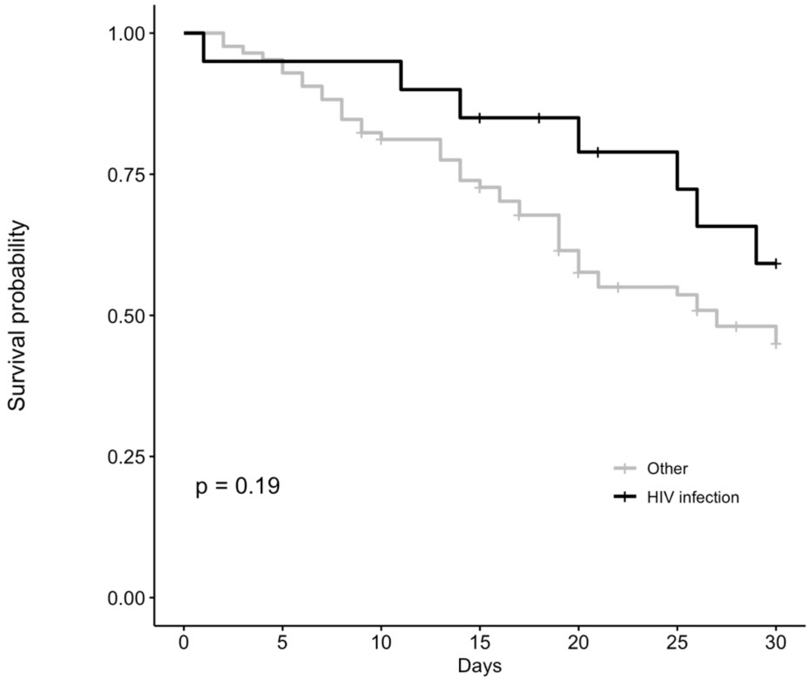

Patients at risk

	0	5	10	15	20	25	30
Other	85	81	69	61	48	40	32
HIV infection	20	19	19	17	14	12	9

Supplementary figure S5 legend. Survival at 30 days in critically ill patients with presumptive/proven *Pneumocystis jirovecii* pneumonia (PJP), stratified according to the presence of human immunodeficiency virus (HIV) infection vs. other baseline diseases/conditions. Follow-up data and HIV status were available for 105/115 patients with presumptive/proven PJP (91%). The time of origin was set at the time of PJP diagnostic workup. Right-censoring was applied at the time of hospital discharge or at 30 days after the time of PJP diagnostic workup, whichever came first. The reported p value is from log-rank test.

Supplementary Figure S6. Survival at 90 days in critically ill patients with PJP, stratified according to the presence of HIV infection vs. other baseline diseases/conditions

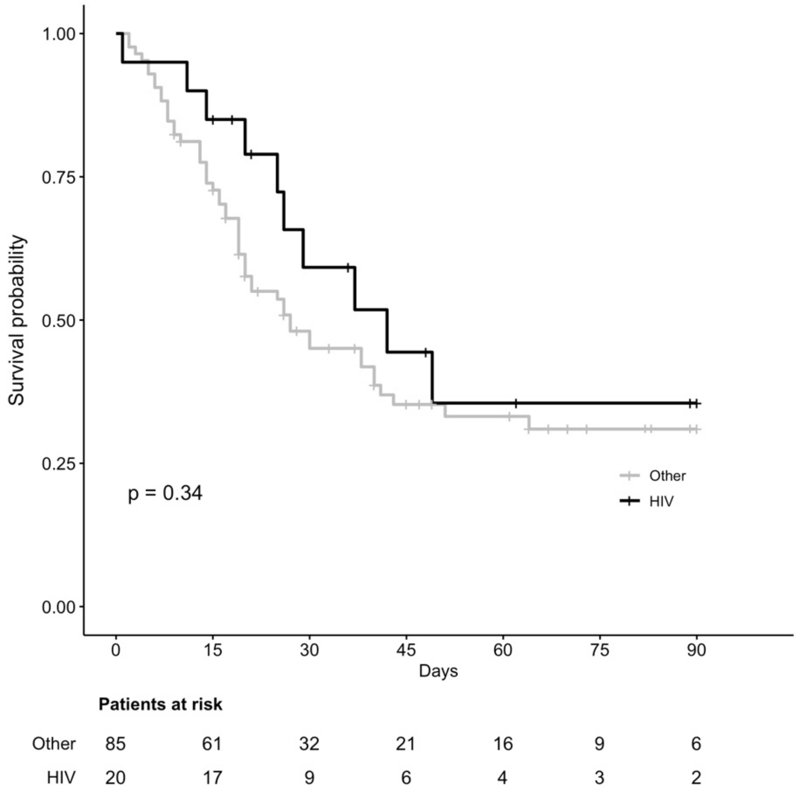

Supplementary figure S6 legend. Survival at 90 days in critically ill patients with presumptive/proven *Pneumocystis jirovecii* pneumonia (PJP), stratified according to the presence of human immunodeficiency virus (HIV) infection vs. other baseline diseases/conditions. Follow-up data and HIV status were available for 105/115 patients with presumptive/proven PJP (91%). The time of origin was set at the time of PJP diagnostic workup. Right-censoring was applied at the time of hospital discharge or at 90 days after the time of PJP diagnostic workup, whichever came first. The reported p value is from log-rank test.
